# Randomized controlled trial of the relative efficacy of high-dose intravenous ceftriaxone and oral cefixime combined with doxycycline for the treatment of *Chlamydia trachomatis* and *Neisseria gonorrhoeae* co-infection

**DOI:** 10.1186/s12879-022-07595-w

**Published:** 2022-07-09

**Authors:** Phuong Thi Thu Nguyen, Ha Viet Pham, Dung Hoang Van, Linh Van Pham, Hoi Thanh Nguyen, Hung Van Nguyen

**Affiliations:** 1 Hai Phong University of Medicine and Pharmacy, 72A Nguyen Binh Khiem, Dang Giang, Ngo Quyen, Hai Phong, Vietnam; 2 Hai Phong International Hospital, 124 Nguyen Duc Canh, Cat Dai, Le Chan, Hai Phong, Vietnam

**Keywords:** *Chlamydia trachomatis*, *Neisseria gonorrhoeae*, Ceftriaxone, Cefixime, Co-infection

## Abstract

**Objectives:**

*Chlamydia trachomatis* (CT) and *Neisseria gonorrhoeae* (NG) are the commonest bacterial causes of sexually transmitted infections in humans with high incidence of co-infection. Treatment with high doses of ceftriaxone (CRO) and cefixime (CFM) is strongly recommended due to the reduced drug susceptibility of NG. However, their safety and efficacy have not been confirmed. We compared the safety and efficacy of a single 1 g intravenous (IV) dose of ceftriaxone (CRO) plus doxycycline (DOX) versus a single 800 mg oral dose of cefixime (CFM) plus DOX for the treatment of NG-CT co-infection.

**Methods:**

An open-label randomized controlled trial was conducted on 125 individuals aged > 18 years with untreated gonorrhea and chlamydia to compare a single 1 g intravenous dose of CRO + DOX and a single 800 mg oral dose of CFM + DOX. The primary outcome was the clearance of NG from all the initially infected sites. Secondary outcomes included symptom resolution, changes in the serum clearance levels, glomerular filtration rate, and antibiotic minimum inhibitory concentrations.

**Results:**

Both regimens were highly effective in treating gonorrhea with success rates of 96.7% (95% confidence interval [CI] 88.8–99.1%) for CRO and 95.3% (95% CI 87.1–98.4%) for CFM. However, CRO + DOX was superior to CFM + DOX for the treatment of NG-CT co-infection (odds ratio 4.41, 95% CI 1.11–25.7). The safety profiles of the two regimens were similar.

**Conclusions:**

CRO + DOX was superior to CFM + DOX for the treatment of NG-CT co-infection. CFM + DOX may be indicated in patients with CRO allergy and in settings where CRO is unavailable.

*Trial registration* ClinicalTrials.gov (NCT05216744) on 31/01/22.

## Introduction

Globally, *Chlamydia trachomatis* (CT) and *Neisseria gonorrhoeae* (NG) are the most common bacterial causes of sexually transmitted infections (STIs) in humans with more than 214 million new infections in 2016 according to the report of the World Health Organization on global sexually transmitted infection surveillance in 2018 [1]. NG not only causes clinical syndromes similar to CT, but also coexists in a significant proportion of patients with chlamydial infection, with up to approximate 40–46% of those with NG infection being simultaneously infected with CT [1, 2].

The definitive diagnosis of gonorrhea is based on a combination of clinical symptoms and laboratory tests, including urethral/vaginal discharge or pus, dysuria, and a history of unprotected sex. Tests for the diagnosis of gonorrhea include Gram staining (urethral or cervical smear), which shows Gram-negative diplococci within and outside the neutrophils [3] If the Gram stain is negative, at least one of two additional cultures or nucleic acid amplification tests is needed to confirm the diagnosis, with culture considered the gold standard for the diagnosis of gonorrhea. Nucleic acid amplification testing (NAAT) is recommended as the optimal method for the diagnosis of genital and extragenital infections caused by NG and CT in patients with or without symptoms [3].

Doxycycline in a dose of 100 mg orally twice daily for 7 days is recommended by the United States Centers for Disease Control and Prevention (CDC) for the treatment of chlamydia in nonpregnant individuals with gonococcal infection [3] Despite the reduced susceptibility of NG, ceftriaxone remains a highly reliable treatment, especially at higher doses [3–5] The CDC guidelines indicate a single dose of ceftriaxone 500 mg intramuscularly (IM) for individuals who weigh < 150 kg and ceftriaxone 1 g IM for those weighing > 150 kg as the first line therapy of uncomplicated gonococcal infection [3] If an injectable ceftriaxone preparation is not available, one oral dose of cefixime 800 mg can be used to treat gonococcal infection. However, a trend toward gradually increasing minimum inhibitory concentrations of both cefixime and ceftriaxone for NG has been observed, indicating a decrease in the drug susceptibility of NG [6, 7] Oral cefixime achieves a microbiological cure in 96% of individuals with uncomplicated gonorrhea. However, the 400 mg cefixime regimen has been shown to be less effective than the IM ceftriaxone regimen [8] Therefore, there have been several recommendations worldwide to increase the dose of cephalosporins to ensure efficacy and reduce resistance [1, 9] The different types of injection including intradermal, subcutaneous, intramuscular, and intravenous injection, which can reach different layers of the skin, thus affecting the level of pain experienced by the patient. While intradermal, intravenous, and subcutaneous injections cause roughly the same amount of pain, intramuscular injections can be more painful in comparison. Indeed, IM injection can cause anxiety and pain in patients, leading to fear of injection and avoidance of repeating this painful experience in patients [10, 11]. Ross et al. [12] reported that 98% (315/320) of gonorrhea patients treated with ceftriaxone IM experienced injection site pain.

The pathogen-pathogen and pathogen-host interactions specific to co-infection may also affect transmission, re-infection, treatment failure, pathogenic resistance, and vaccine development, which may ultimately influence decisions regarding the choice of medical therapy and treatment efficacy [13]. Although numerous studies have investigated different aspects of these issues, the reported findings are often contradictory. Therefore, we performed a non-blind randomized controlled clinical trial to compare the efficacy of two combination high-dose cephalosporin therapies, including ceftriaxone (1 g IV) + doxycycline and cefixime (800 mg PO) + doxycycline in the treatment of *N. gonorrhoeae* and *C. trachomatis* co-infection.

## Methods

### Participants and study design

The study protocol was reviewed and approved by the Hai Phong International Hospital Institutional Review Board. The study was conducted in accordance with the Declaration of Helsinki and International Conference on the Harmonization of the Technical Requirements for the Registration of Pharmaceuticals for Human Use—Good Clinical Practice guidelines and the guideline for Vietnamese Good Clinical Practice. Written informed consent was obtained from all participants before study initiation. This trial was registered with ClinicalTrials.gov (NCT05216744) on 31/01/22.

This study was a single-center, parallel-group, randomized controlled trial that compared treatment with 1 g of ceftriaxone IV to treatment with oral cefixime together with doxycycline in patients with NG and CT co-infection. This study was conducted in The Department of Nephrology – Urology of Hai Phong International Hospital from 1 October 2021 to 02 February 2022.

Individuals aged > 18 years were eligible for participation if they had a diagnosis of untreated gonorrhea and chlamydia. The untreated status was defined as no antibiotic taken in the previous 28 days to treat gonorrhea and chlamydia, either partially or completely. Diagnosis was based on the detection of NG and CT by NAAT from the first voided urine or urethral, endocervical, vulvovaginal, pharyngeal, or rectal swabs.

Exclusion criteria were known contraindications or hypersensitivity to cephalosporins, penicillins, or doxycycline; gonorrhea with complications, such as pelvic inflammatory disease or epididymo-orchitis; and significant renal failure or hepatic failure. Women who were pregnant or breastfeeding were excluded. Significant renal failure is defined as the presence of decreased kidney function (defined as estimated glomerular filtration rate [eGFR] < 30 mL/min/1.73 m^2^). Interventions will not be initiated if the patient exhibits active liver disease or increased transaminases (ALT > 2.5 times the upper limit of normal) at baseline.

### Randomization and intervention

Within 24 h of admission, patients were randomly assigned to one of two groups using a computer-generated list of random numbers, with one group taking a regimen of 1 g of ceftriaxone IV and the other receiving one oral dose of 800 mg cefixime. All participants were administered 100 mg of doxycycline orally twice a day for 7 days. For 1 g ceftriaxone intravenous injection, ceftriaxone was dissolved in 10 mL of water for injections and then injected over 5 min, directly into the vein of participants.

Safety information was recorded daily with the assistance of a clinical pharmacist to document any adverse events. All participants were asked to abstain from sex until the end of the study.

### Outcomes and follow-up

Patients were clinically evaluated at two time-points, including day 1 (before drug administration) and day 8 (end of the 7-day treatment) by nephrologist-urologists. Clinically reported symptoms included genital discharge, dysuria, sore throat, anorectal pain, rectal bleeding, rectal discharge, and constipation. In general, NAAT and culture are the tests of choice for the microbiologic diagnosis of NG and CT infection. Samples were collected from the participants for the detection of NG and CT before treatment initiation and on the day 8 of the study for NAAT and cultures. The samples were selected according to the sex and sexual orientation of the participant: urethral samples from heterosexual men; urethral, pharyngeal, and rectal samples from men who have sex with men; cervical, pharyngeal, and rectal samples from women. NAAT and cultures were performed on the samples from the cervix, pharynx, and rectum. Follow-up was performed 1 week after treatment, when NAAT and cultures for NG were repeated for sites that had been positive at the baseline. All pre- and post-treatment samples were subjected to NAAT (Aptima Combo 2, Hologic, MA, USA). The identification of NG by culture was performed according to guidelines of the CDC [14]. The E-test method (Durham, NC, USA), was applied to determine the minimum inhibitory concentration (MIC) of ceftriaxone, and cefixime for the NG isolates. Whole blood samples were obtained to assess the renal function of patients in both the groups on the first and last day of the study.

The primary endpoint was the clearance of NG from all the initially infected sites, which was defined as a negative NAAT after 1 week of treatment [3]. Success was defined as the eradication of NG and CT (absence of the original pathogens in adequate samples taken after the completion of therapy). Failure was defined as persistence (continued presence of the original bacterial infection upon completion of treatment).

The secondary endpoints included the resolution of the clinical symptoms, including genital discharge, dysuria, sore throat, anorectal pain, rectal bleeding, rectal discharge, and constipation as well as changes in the serum clearance levels, estimated glomerular filtration rate, and antibiotic minimum inhibitory concentration (MIC).

### Sample size and statistical analyses

The sample size was estimated based on the primary outcomes of a trail by Muratani et al. [15], that had a 100% treatment success rate in patients treated with 1 g of ceftriaxone IV, and a trial by Allen et al. [16], that had a treatment success rate of 93% in patients treated with cefixime. In total, 114 patients were required in both arms of the study to compare the effects of 1 g ceftriaxone IV therapy and 800 mg PO cefixime therapy in combination with doxycycline 100 mg twice daily on day 8 with 80% statistical power at a 5% level of significance.

All analyses were performed using SAS (version 9.4, SAS Institute, Cary, NC, USA) and R software, version 3.2.4 [17]. The Independent samples t-test was selected to compare the means between two independent groups on a continuous variables with normal distribution. The Mann–Whitney U test was used to compare differences between two group of non-normally distributed data. Fisher’s exact test was performed to analyzed the difference in proportions between the two categories [18]. There were no missing data.

## Results

**Fig. 1:**
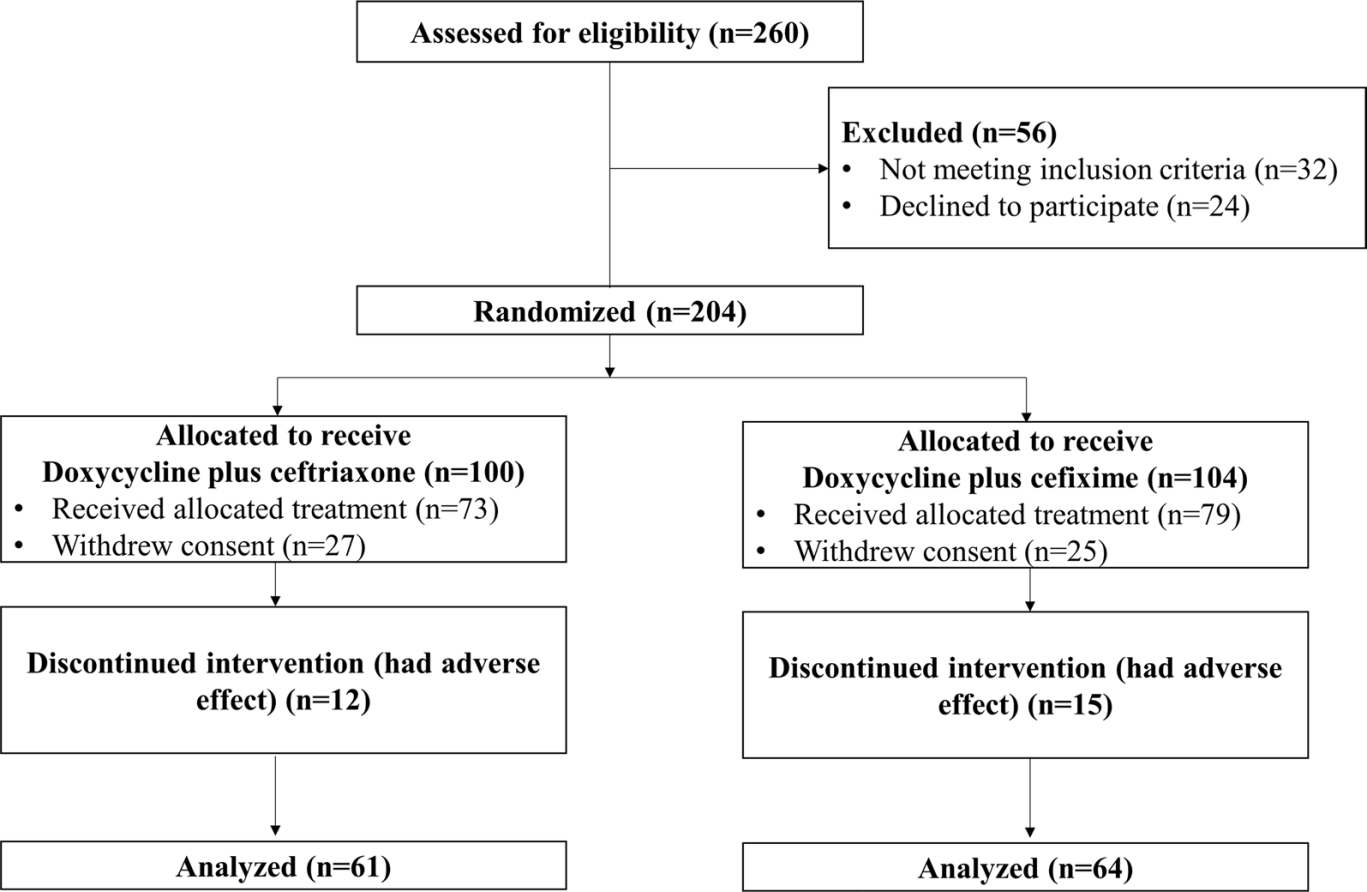
Study design

Of the 260 participants screened, 204 were enrolled and randomly assigned to either the ceftriaxone plus doxycycline group (100 subjects), or cefixime plus doxycycline (104) group. The majority of participants in the ceftriaxone (61 of 100) and cefixime (64 of 104) groups completed the trial (Fig. 1). Withdrawal of consent was the most common cause for discontinuing participation in the study. Table 1 shows the clinical characteristics of the patients according to group. The patients in the ceftriaxone and cefixime groups were similar in age and body mass index (BMI), and the majority of participants in both groups were male.

**Table 1:** Patient characteristics

	Doxycycline + ceftriaxone (n = 61)	Doxycycline + cefixime (n = 64)	p
Age (mean (SD))	30 ± 6.98	34.61 ± 10.19	0.282
BMI (mean (SD)	23.64 ± 3.2	22.6 ± 2.83	0.17
Sex (male) (n (%))	58 (95.08)	59 (92.19)	0.718
Medical history (n)			
Diabetes	2	1	0.613
Hypertension	1	2	1
Renal disease	2	9	0.055
Liver disease	11	20	0.101
Sites of infection (n)			
Genital	36	41	0.586
Pharyngeal	12	7	0.216
Rectal	4	6	0.744
Genital + pharyngeal	8	6	0.578
Rectal + pharyngeal	1	4	0.366

The two groups had similar comorbidities, including diabetes, hypertension, kidney disease, and liver disease. Similarly, there were no statistically significant differences in the sites infected by NG and CT, including genital, rectal, and pharyngeal sites between the two groups of patients using ceftriaxone + doxycycline and cefixime + doxycycline.

The combination therapy of 1 g ceftriaxone IV plus doxycycline had a high cure rate of 95.1% (95% confidence interval [CI] 86.5–98.3) for NG and CT co-infection. A single oral dose of 800 mg of cefixime combined with doxycycline PO had a cure rate of 81.2% (52/64; 95% CI 70.0–88.9) after 7 days of treatment. Thus, doxycycline + ceftriaxone therapy was superior to cefixime plus doxycycline therapy for eradicating CT and NG (odds ratio [OR] 4.41, 95% CI 0.6–10.91, p = 0.026). However, the two therapies did not differ significantly in their ability to eradicate either NG or CT separately (OR for bacterial clearance 0.27 [95% CI 0.6–16.8] and 1.45 [95% CI 0.16–17.89], respectively (Table 2).

**Table 2:** Clearance of *Neisseria gonorrhoeae* and *Chlamydia trachomatis* at all infected sites at 8th day

Infected sites	Doxycycline + ceftriaxone (n = 61)		Doxycycline + cefixime (n = 64)		ORs (95% CI)	p value
	n	% (95% CI)	n	% (95% CI)		
Subjects eradicated CT						
Genital	36	100.0	41	100.0		1
Pharyngeal	12	100.0	4	57.1		0.036
Rectal	4	100.0	6	100.0		1
Genital + pharyngeal	6	75.0	4	66.7	1.46 (0.1–28.6)	1
Rectal + pharyngeal	0		1	25.0		1
All sites	58	95.1 (86.5–98.3)	56	87.5 (77.2–93.5)	2.7 (0.6–16.8)	0.21
Subjects eradicated NG						
Genital	36	100.0	41	100.0		1
Pharyngeal	12	100.0	6	85.7		0.37
Rectal	4	100.0	6	100.0		1
Genital + pharyngeal	6	75	5	83.3	0.6 (0.1–15.5)	1
Rectal + pharyngeal	1	100.0	3	75.0		1
All sites	59	96.7 (88.8–99.1)	61	95.3 (87.1–98.4)	1.45 (0.16–17.89)	1
Subjects eradicated CT and NG						
Genital	36	100.0	41	100.0		1
Pharyngeal	12	100.0	3	42.9		0.009
Rectal	4	100.0	5	83.3		1
Genital + pharyngeal	6	75.0	2	33.3	5.2 (0.4–108.1)	0.28
Rectal + pharyngeal	0	0.0	1	25.0		1
All sites	58	95.1 (86.5–98.3)	52	81.2 (70.0–88.9)	4.41 (1.11–25.7)	0.026

*CT Chlamydia trachomatis*; *NG Neisseria gonorrhoeae*

**Fig. 2:**
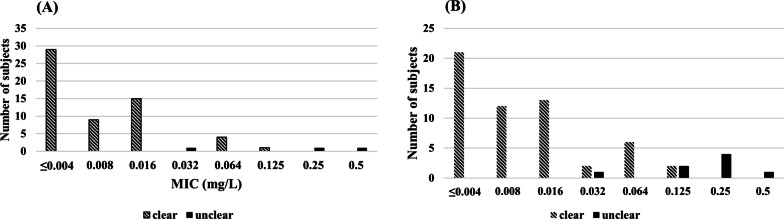
Distribution of the baseline MIC of ceftriaxone and cefixime in 125 patients who were assigned to use either ceftriaxone (**A**) or cefixime (**B**). *MIC* minimum inhibitory concentration

For the 310 specimens from 260 patients, the positive rate of the NG cell culture test was 49.5% while that of the NAAT test was 75.2% (p < 0.001). In the group of patients who cleared NG from all sites of infection, 29 samples had ceftriaxone MIC < 0.004, 13 patients had ceftriaxone MIC = 0.008, and only two patients had ceftriaxone MIC = 0.125. Specifically, a significantly lower proportion of patients in the NG clear group (2/28 patients) had a ceftriaxone MIC ≥ 0.125 compared to the NG treatment failure group (7/8 patients) (p < 0.001), as shown in Fig. 2. Similar observations were made in the cefixime group where the treatment success group had a smaller MIC than the failure group.

While looking at the resolution of symptoms of NG and CT infection in our study groups, we found that the two therapies were equally effective in relieving most symptoms such as painful urination, sore throat, anorectal pain, rectal bleeding, rectal discharge, and constipation (p > 0.05). However, doxycycline + ceftriaxone therapy was more likely to resolve genital discharge symptoms than doxycycline + cefixime therapy (p = 0.017) (Table 3).

**Table 3:** Resolution of symptoms present at baselines

	Doxycycline + ceftriaxone (n = 61)^#^	Doxycycline + cefixime (n = 64)^#^	OR (95% CI)	p value
Genital discharge	49/56	31/46	3.35 (1.13–10.86)	0.017
Dysuria	35/43	31/40	1.27 (0.38–4.29)	0.787
Sore throat	18/26	16/25	1.26 (0.34–4.78)	0.771
Anorectal pain	20/26	20/28	0.95 (0.24–3.9)	1
Rectal bleeding	13/17	24/26	0.28 (0.02–2.25)	0.193
Rectal discharge	9/10	6/8	0.35 (0.01–8.27)	0.559
Constipation	9/13	11/12	0.2 (0–2.48)	0.322

^#^Number of patients at baseline/number of patients at day 8

The two therapies evaluated in our study had similar safety and toxicity profiles, including similar incidence of skin rash, eosinophilia, leukopenia, increased serum transaminases, abdominal distention, diarrhea, abdominal pain, and vomiting with p-values > 0.05 (Table 4). We also recorded 6/61 cases of pain at the injection site in the doxycycline + ceftriaxone group, along with one case with redness at the injection site of ceftriaxone.

**Table 4:** Recorded side-effects in study

	Doxycycline + ceftriaxone (n = 61), (n (%))	Doxycycline + cefixime (n = 64), (n (%))	OR (95% CI)	p value
Skin rash	3 (4.92)	1 (1.56)	3.23 (0.25–173.57)	0.357
Eosinophilia	2 (3.28)	3 (4.69)	0.69 (0.06–6.26)	1
Leukopenia	2 (3.28)	3 (4.69)	0.69 (0.06–6.26)	1
Increased serum transaminases	4 (6.56)	2 (3.13)	2.16 (0.3–24.77)	0.432
Abdominal distention	1 (1.64)	3 (4.69)	0.34 (0.01–4.39)	0.619
Diarrhea	3 (4.92)	7 (10.94)	0.42 (0.07–1.97)	0.325
Abdominal pain	2 (3.28)	6 (9.38)	0.33 (0.03–1.95)	0.274
Vomiting	1 (1.64)	4 (6.25)	0.25 (0–2.65)	0.366
Pain at injection site	6 (9.84)			
Tenderness at injection site	1 (1.64)			

The serum clearance levels before and after 7 days of treatment in both the doxycycline + ceftriaxone and doxycycline + cefixime therapy groups were not statistically different (p > 0.05). However, we found that the pre-treatment glomerular filtration rate (GFR) was slightly higher than the GFR after 7 days of treatment in the doxycycline + cefixime treatment group (100.22 ± 26.31 vs 93.38 ± 20.92 mL/min/1.73 m^2^, p = 0.047), and this finding was not observed in patients receiving doxycycline + ceftriaxone (Table 5).

**Table 5:** Changes in serum clearance and Glomerular filtration rate

	Doxycycline + ceftriaxone	p value	Doxycycline + cefixime	p value	p value
Clearance (mmol/L)					
At baseline	86.98 ± 40.26	0.661	80.32 ± 19.54	0.217	0.238
At 8th day of treatment	84.57 ± 17.89		83.63 ± 15.44		0.752
GFR (mL/min/1.73 m^2^)					
At baseline	99.83 ± 30.03	0.176	100.22 ± 26.31	0.047	0.939
At 8th day of treatment	95.33 ± 18.73		93.38 ± 20.92		0.586
Delta clearance	− 2.28 ± 40.35		3.31 ± 21.28		0.331
Delta GFR	− 4.54 ± 25.91		− 6.84 ± 26.7		0.626

*GFR* glomerular filtration rate

## Discussion

Our study results showed that an oral dose of 800 mg cefixime was inferior to a single IV dose of 1 g of ceftriaxone for the treatment of gonorrhea and chlamydia co-infection when both drugs were taken together with oral doxycycline 100 mg twice a day for 7 days. The 13.9% greater clearance of infection in the ceftriaxone group compared to the cefixime group as well as the consistency of results on MIC analyses indicate that IV ceftriaxone is more effective than PO cefixime for the microbiological cure of gonorrhea and chlamydia. However, when considering the possibility of pathogenic eradication of CT or NG separately from all sites of infection, ceftriaxone and cefixime showed comparable efficacy. In terms of the alleviation of clinical symptoms, we found that the two therapies had similar effects on symptoms such as dysuria, sore throat, anorectal pain, rectal bleeding, rectal discharge, and constipation However, IV ceftriaxone at a dose of 1 g demonstrated better potential in reducing genital discharge symptoms than cefixime in our study.

Although high-dose ceftriaxone is strongly recommended for the treatment of gonorrhea because of its reduced susceptibility, the evidence available from clinical studies is still limited. British Association for Sexual Health and HIV (BASHH) in the United Kingdom [19], China [20, 21], and Japan [22] recommended that ceftriaxone should be administrated by both IM and IV routes for uncomplicated *N. gonorrhoeae* infections of the urethra, cervix, rectum, and pharynx in adults and youth. A clinical study in Japan evaluating the effectiveness of 1 g of ceftriaxone demonstrated a cure rate of 100% in 48 patients with urethral infection or cervical gonococcal infection including infection with strains with chimera penicillin binding protein 2 (PBP-2) expression that are resistant to oral cephalosporins [15] A systematic review and meta-analysis of clinical trials that investigated the efficacy of 800 mg of PO cefixime for the treatment of gonorrhea of the urethra, cervix, or rectum reported a cure rate of 98.0% [23]. A meta-analysis of 23 randomized trials concluded that the microbial cure rates were slightly higher with doxycycline than azithromycin (97.4% vs 96.2%) for the treatment of uncomplicated genital *C. trachomatis* infections [24] Our study results revealed that the cure rate for CT in both groups was 114/125 (91.2%), which was slightly lower than the results of the abovementioned study. This may be because the timing of our bacteriological eradication evaluation was immediately after the end of the study (day 8), whereas in other studies it was 1 week after stopping the doxycycline therapy.

Although the prevalence of NG and CT co-infection is very high, there is a significant gap in our knowledge of the acquired factors, pathogenesis, load, severity, treatment, and post-treatment follow-up. A single-center observational study in Japan on a cohort of men who have sex with men indicated that the efficacy of dual therapy consisting of 1 g of ceftriaxone IV and a single oral dose of 1 g azithromycin or 100 mg doxycycline to treat extragenital NG infection was 95.5% (107/112, 95% CI 90.0–98.1%) [25]. We could not find any clinical studies comparing ceftriaxone 1 g IV and cefixime 800 mg in combination with oral doxycycline for the treatment of NG and CT co-infection. A randomized, unblinded multicenter study including 209 men and 124 women with uncomplicated gonorrhea showed the comparable efficacy of single-dose regimens of 800 mg cefixime and 250 mg IM ceftriaxone with cure rates of 98% (95% CI 94.6%–100%) and 98% (95% CI 94.9%–100%), respectively [26]. While CT infection was detected in at least half of the infected patients in each treatment group, the study did not evaluate the effect of the treatment on this infection. Recently, ceftriaxone has been shown to be more effective than oral cephalosporins. In a meta-analysis of trials evaluating the treatment of uncomplicated gonorrhea, a higher cure rate was reported with ceftriaxone 250 mg IM compared to cefixime 400 mg (OR 1.77) with comparable adverse events [8].

The adverse events observed with both therapies, including skin rash, eosinophilia, leukopenia, increased serum transaminases, abdominal distention, diarrhea, abdominal pain, vomiting did not show a statistically significant difference. A systematic review and meta-analysis of four comparative trials reported that the occurrence of at least one adverse drug reaction did not differ significantly between the cefixime and ceftriaxone groups [27]. Handsfield et al. [26] revealed that the most frequently reported adverse effects of ceftriaxone (250 mg IM) and a single dose of cefixime (400 or 800 mg orally) were gastrointestinal in nature, specifically diarrhea and nausea. Our study found no difference in the blood clearance levels after 7 days of treatment for gonorrhea and chlamydia in both the treatment groups. However, in the group of patients using cefixime and doxycycline, there was a slight but significant decrease in the GFR. Since the treatment effects were evaluated on day 8 of treatment, there are limitations regarding monitoring of the long-term efficacy and safety of both therapies. However, according to the CDC guidelines, a test of cure can be performed 7–14 days following the treatment. Test of cure is not routinely warranted. Pregnancy, patients with persistent symptoms, nonadherence to the regimen, use of a regimen with inferior cure rates, such as erythromycin or amoxicillin, azithromycin treatment of patients with or at high risk for rectal infection should be performed no sooner than 4 weeks after treatment. This is especially important when NAATs are used because C. trachomatis nucleic acid may still be tested positive on at least one of the samples taken after 3 weeks [14, 28, 29]. However, our subjects who are not at risk for developing the false positive NAAT test after antibiotic treatment. Evaluation of the efficacy and safety of antibiotic therapy for the treatment of gonorrhea and chlamydia is essential for optimal clinical decision [3]. Our study did not perform cultures tests for chlamydia because the accuracy of cell culture is less than that of NAAT. However, the sensitivity of cell culture is more than that of NAAT [30]. The previous study indicated that NAAT should be considered the new “gold standard” for the laboratory diagnosis of CT infections.

## Conclusion

In conclusion, we found that treatment with ceftriaxone 1 g IV plus doxycycline was effective with a high cure rate for NG and CT co-infection. This combination therapy was found to be superior to cefixime 800 mg plus doxycycline, with a relatively higher frequency of treatment success in patients with gonorrhea and chlamydia co-infection. However, cefixime 800 mg together with doxycycline achieved a high cure rate for gonorrhea, and its indication could be appropriate in individuals with a history of allergy or intolerance to ceftriaxone. Patients in the successful treatment group had significantly lower MICs for ceftriaxone and cefixime than in the failure group for the microbiological targets.

## Data Availability

The data that support the findings of this study are available from Hai Phong International Hospital but restrictions apply to the availability of these data, which were used under license for the current study, and so are not publicly available. Data are however available from the authors upon reasonable request and with permission of Hai Phong International Hospital.
